# Prediction of pelvic tumour coverage by magnetic resonance-guided high-intensity focused ultrasound (MRgHIFU) from referral imaging

**DOI:** 10.1080/02656736.2020.1812736

**Published:** 2020-09-01

**Authors:** Ngo Fung Daniel Lam, Ian Rivens, Sharon L. Giles, Emma Harris, Nandita M. deSouza, Gail ter Haar

**Affiliations:** a Joint Department of Physics, The Institute of Cancer Research, London, UK; b The CRUK Cancer Imaging Centre, The Institute of Cancer Research and The Royal Marsden NHS Foundation Trust, London, UK

**Keywords:** Treatment planning, magnetic resonance imaging guidance, high intensity focused ultrasound, human body deformation, pelvis, referral imaging

## Abstract

**Background:**

Patient suitability for magnetic resonance-guided high intensity focused ultrasound (MRgHIFU) ablation of pelvic tumors is initially evaluated clinically for treatment feasibility using referral images, acquired using standard supine diagnostic imaging, followed by MR screening of potential patients lying on the MRgHIFU couch in a ‘best-guess’ treatment position. Existing evaluation methods result in ≥40% of referred patients being screened out because of tumor non-targetability. We hypothesize that this process could be improved by development of a novel algorithm for predicting tumor coverage from referral imaging.

**Methods:**

The algorithm was developed from volunteer images and tested with patient data. MR images were acquired for five healthy volunteers and five patients with recurrent gynaecological cancer. Subjects were MR imaged supine and in oblique-supine-decubitus MRgHIFU treatment positions. Body outline and bones were segmented for all subjects, with organs-at-risk and tumors also segmented for patients. Supine images were aligned with treatment images to simulate a treatment dataset. Target coverage (of patient tumors and volunteer intra-pelvic soft tissue), i.e. the volume reachable by the MRgHIFU focus, was quantified. Target coverage predicted from supine imaging was compared to that from treatment imaging.

**Results:**

Mean (±standard deviation) absolute difference between supine-predicted and treatment-predicted coverage for 5 volunteers was 9 ± 6% (range: 2–22%) and for 4 patients, was 12 ± 7% (range: 4–21%), excluding a patient with poor acoustic coupling (coverage difference was 53%).

**Conclusion:**

Prediction of MRgHIFU target coverage from referral imaging appears feasible, facilitating further development of automated evaluation of patient suitability for MRgHIFU.

## 1. Introduction

Magnetic resonance guided high-intensity focused ultrasound (MRgHIFU) is a noninvasive, non-ionizing treatment modality which has a number of established clinical applications including the ablation of uterine fibroids and bone nerves (for pain palliation) [1], and the treatment of essential tremor [2]. In addition, MRgHIFU is being trialed in the UK for the thermal ablation of recurrent gynaecological tumors (NCT02714621) [3].

MRgHIFU therapy of pelvic tumors is particularly challenging because of the depth of the tumors within the body. MRgHIFU systems can only treat targets within the focal length constraints of their transducers, and identifying acoustic access which is free from obstruction by acoustically opaque tissues, such as gas and bone, and from organs at risk is challenging [3]. Failure to correctly identify suitable patients for MRgHIFU therapy could deprive them of their only treatment option, while failure to identify patients who cannot be treated wastes patient time and hospital resources on screening sessions. Patients must therefore be carefully assessed prior to being accepted for treatment. We hypothesize that an algorithm could be developed, that could accurately predict target tumor coverage by HIFU from referral imaging.

Currently, the clinical evaluation process relies heavily on experience and opinion. The process is as follows: patients are referred to the MRgHIFU clinic on the basis of supine diagnostic imaging, often follow-up imaging after unsuccessful prior treatment [3,4] and referred to here as the ‘referral image dataset’. If at this point treatment appears qualitatively feasible, patients progress to the screening stage. At screening, patients are imaged with treatment conditions being mimicked as closely as possible. Patients are asked to lie in one or two ‘best guess’ treatment positions on the MRgHIFU couch. The ‘best guess’ positions are identified by the treatment team using prior clinical experience and subjective judgment. Suitable patients, those for whom a majority of the tumor can be reached or who fulfill clinical trial eligibility criteria, are invited back for treatment. The current process is challenging. In a previous metastatic bone pain palliation trial, 16 of 37 patients (43%) initially considered for treatment were found at screening not to satisfy eligibility criteria because of disease that could not be targeted, for reasons that include tumor accessibility and size [4]. In a pilot planning study which assessed MRgHIFU for the treatment of recurrent gynaecological tumors, 9 of 20 eligible patients (45%) who underwent screening imaging were subsequently assessed as untreatable because of an eligibility criterion, namely, that >50% tumor coverage could be achieved without risk of damage to surrounding structures [3]. These two studies suggest that, for abdominal pelvic tumors, the current evaluation process may overestimate the number of patients that are suitable for MRgHIFU by more than 40%.

**Figure 1.**
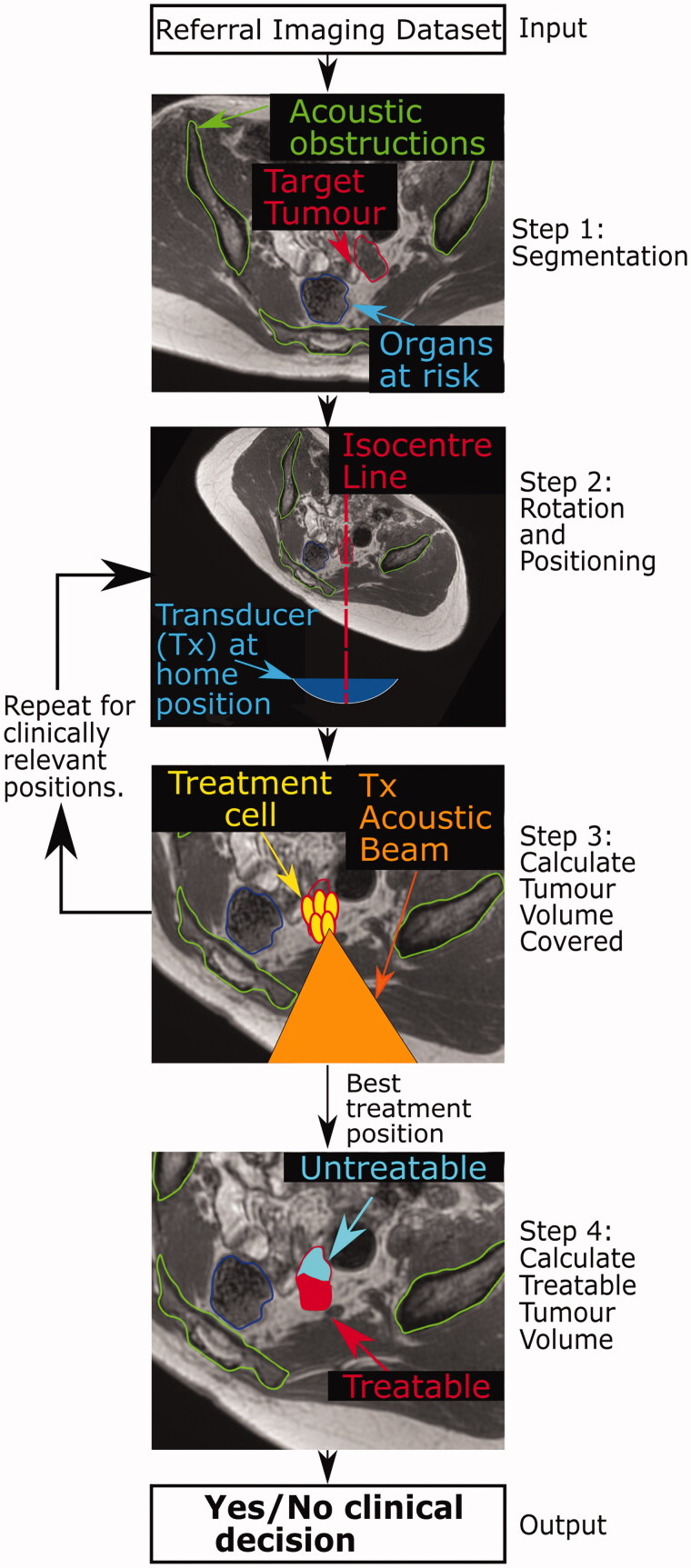
Schematic of proposed patient workflow. Workflow designed to assess the potentially MRgHIFU-treatable percentage of a patient’s target tumor. Using a supine referral image dataset, step 1 involves segmentation of important structures: organs at risk, acoustic obstructions, and the target tumor. Step 2 rotates the referral imaging dataset into possible treatment positions, with the tumor centroid lying, by idealized design, along a vertical line through the magnetic isocentre and, by system design, the transducer’s home position. In step 3, target coverage (i.e. percentage of target volume coverable by an 8 mm treatment cell) is calculated. Cycling through steps 2 & 3 identifies the patient orientation with the maximum target volume coverage. In step 4, the treatable percentage of the target volume is quantified, using acoustic and thermal modeling of MRgHIFU treatment. This allows a clinical decision of whether to progress to treatment to be made.

Given the relatively poor results of the current subjective method, we propose a workflow that would ultimately be suitable for the quantitative assessment of patient suitability for MRgHIFU therapy (Figure 1). In this paper, we focus on a core aspect of that workflow, as explained below. If the workflow were to be successfully implemented, the number of patients incorrectly denied treatment could be minimized, and the number who would benefit from a screening scan could be maximized. In the long-term, it may even be possible to avoid the need for a screening visit, which could mean that a sick patient will no longer need to travel to the magnetic resonance (MR) imaging unit and undergo what may be a lengthy session in which optimal treatment positions are investigated, only to return days to weeks later for a treatment session. This may also reduce the load on the resources of a busy clinical MR department.

The proposed patient workflow (Figure 1) comprises three steps. In Step 1, key anatomical components that could prevent access to targets, such as acoustic obstructions and organs at risk, are segmented from the referral images. In Step 2, the referral imaging dataset is orientated into plausible potential treatment positions. In Step 3, the percentage of tumor volume that can be reached by the HIFU focus (% target volume covered) is calculated at each orientation. In Step 4, acoustic and thermal modeling are used to calculate the treatable target volume, in order to facilitate a quantitative clinical decision as to whether a patient should proceed to screening.

The focus of this paper is Step 3, the calculation of tumor coverage. As far as the authors are aware, no previous work has been done on predicting target tumor coverage from referral images. A novel method has been developed to identify the tumor coverage that could be achieved in the presence of acoustic obstructions and organs at risk, and using this methodology, a feasibility study has been performed to determine whether it is possible to accurately predict tumor coverage from referral imaging by comparison with predictions made using subjects lying in treatment orientations. For this purpose, volunteer imaging data were obtained, and used to develop novel data processing and analysis techniques for the calculation of tumor coverage. Subsequently, the method was tested using patient data obtained in a concurrently started clinical trial.

## 2. Methods

### 2.1. Overview

**Figure 2.**
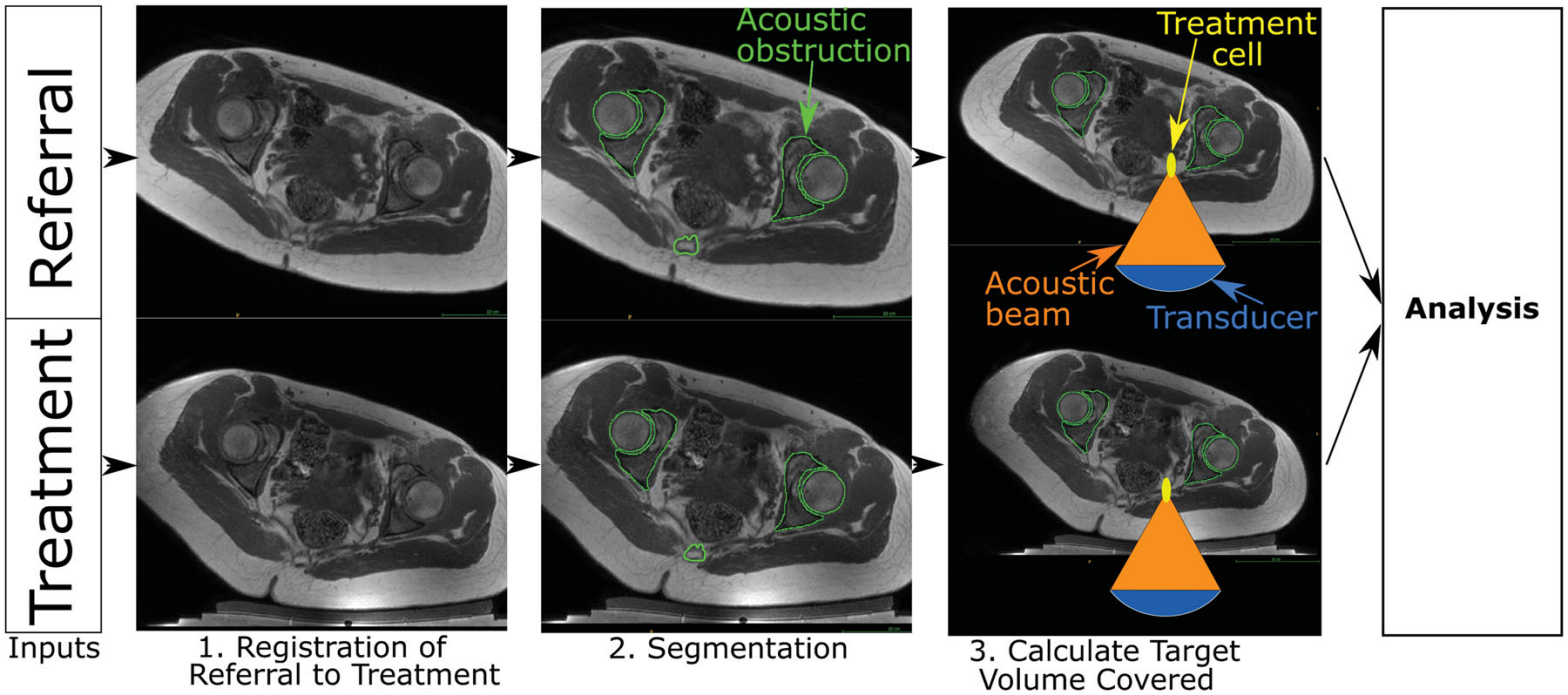
Schematic of developmental methodology used in this study. The accuracy of the methodology to calculate target coverage from referral imaging was assessed using this workflow. The target volume coverage by MRgHIFU was calculated from a subject’s treatment image dataset, acquired with the subject placed in a plausible or actual treatment position (bottom row) for volunteers or patients, respectively. Comparison with the target volume coverage predicted from a supine referral image datasetet allowed assessment of the methodology. Step 1: the referral imaging dataset is rotated into the same orientation as the treatment imaging dataset using affine registration both to allow comparison with the treatment imaging dataset. Step 2: segmentation of acoustic obstructions (e.g. bones, shown), organs at risk (patients only) and the target tumors (patients only) was performed to identify tissues that impede target coverage. Step 3: Target volume coverage was calculated for the registered-referral imaging dataset and the treatment imaging dataset, and finally, the two quantities were compared to assess the predictive capacity of the methodology.

In order to evaluate the developed methodology for the calculation of tumor coverage, estimations of target (tumor) coverage from referral and treatment images obtained for each volunteer (patient) were compared. Here, the referral imaging dataset is the expected input into the prospective patient workflow and is used to predict target volume coverage. We assume the treatment images depict the subject positioned in a plausible (volunteer) or actual (patient) treatment position, respectively, on the MRgHIFU bed. The treatment imaging dataset is used to calculate the target coverage. The workflow used in this study is shown in Figure 2. As the treatment position is known from the treatment images, the referral imaging dataset was oriented into the known treatment position to compare the predicted target coverage with the actual target coverage. This was achieved by an affine registration of the referral imaging dataset to the treatment imaging dataset (Step 1 in Figure 2). Segmentation of the acoustic obstructions and organs at risk (Step 2 in Figure 2) from both datasets was performed to identify tissues that could prevent target coverage. This was followed by calculation of the target (tumor) coverage (Step 3 in Figure 2) and comparison of the results for predictions from referral imaging datasets with those from treatment imaging datasets.

At the start of the project, clinical trial data were not available. The method was therefore developed using volunteer imaging data, with the goal of testing it on anticipated clinical datasets. As a result of significant anatomical differences between volunteers and patients, some adaptation was necessary. Firstly, volunteers lacked target tumors. This could have been addressed by the creation of dummy tumors, but in the absence of an obvious method for defining the size, shape and position of dummy tumors in an unbiased and clinically relevant way, all the soft tissue in the pelvis was defined as ‘target tissue’. Secondly, while patients undergo dietary and physical bowel preparation prior to treatment in order to minimize the risk of bowel and rectal damage, volunteers were not required to do so. These tissues were therefore not considered to be organs-at-risk when processing volunteer data. While these two limitations present challenges, they do not prevent like-for-like comparison between target coverage predictions from referral and treatment imaging datasets. Datasets from 5 volunteers, comprising pseudo-referral and pseudo-treatment imaging datasets were available for the development of the method. The methodology was subsequently tested on 5 patients who had undergone ablative MRgHIFU treatment for recurrent gynaecological tumors.

### 2.2. Input images

All subjects were scanned on a 3.0 T Philips Achieva^®^ MR scanner (Amsterdam, Netherlands), using a multi-point Dixon sequence [5] (TE1/TE2 = 1.186 (out-of-phase)/2.372 (in-phase) ms, TR = 3.62 ms, number of echoes = 2, flip angle = 10°). This produced four 3D image sets for each referral and treatment imaging dataset: in-phase (`IP'), out-of-phase (`OP'), water-only (`Water') and fat-only (`Fat') image sets. Patients were further imaged using, amongst others, a T2w Large Field-of-View (T2wLFOV) sequence.

All referral imaging datasets were acquired with subjects lying supine on the standard MR bed using SENSE XL torso coils (Philips, Netherlands) wrapped around the pelvis. Treatment imaging datasets were acquired with subjects lying oblique supine decubitus on a gel-pad, which was placed on top of an acoustically transparent membrane on the top surface of the Sonalleve^®^ V2 MRgHIFU couch (Profound Medical, Mississauga, Canada), using two Sonalleve^®^ coils – one integrated into the acoustic window, and an external pelvic coil. The subject’s body weight caused the gel-pads to compress and the membrane to bow. Subjects were positioned by a radiographer experienced in MRgHIFU. Cohort-specific imaging information for volunteers is given in Section 2.2.1, and for patients, in Section 2.2.2.

Treatment angles were measured using ITK-Snap 3.6.0 software [6] (University of Pennsylvania, USA), by manually drawing a line between the axial-plane positions of the left and right ischial spines, and finding the angle between this and a horizontal line.

#### 2.2.1. Volunteers

Five female volunteers (age: 28–44 years, weight: 55–72 kg, body mass index: 20.2–26.4 kg/m^2^), were scanned (with ethics approval from The Royal Marsden and ICR Committee for Clinical Research (internal protocol CCR1406)). In addition to the supine referral imaging dataset described above, each volunteer was scanned in two ‘treatment’ positions deemed to be plausible from experience of treating patients with pelvic bone pain with MRgHIFU [3,4]. These positions were nominally `steep' and `shallow', but were dependent on a subject’s size and shape, which affected how they fitted into the bore of the MR scanner. This generated two treatment imaging datasets per volunteer. The volunteers, wearing thin trousers, were placed with their left buttock roughly centered over the acoustic window and with their right side elevated using angled foam pads. They were scanned from the L5-sacrum disk to the inferior-most point of the ischial tuberosity in the axial direction. Fields-of-view were chosen to include the full body outline in the axial slices. 15 mm-thick gel-pads were used to provide acoustic coupling between the skin and the Sonalleve^®^ acoustic window for all volunteers. The voxel size for referral imaging and treatment imaging datasets was approximately 0.78 × 0.78 × 1.50 mm^3^. Volunteer details are recorded in Table 1.

**Table 1.** Details of volunteers participating in this study.

Volunteer	1	2	3	4	5	Mean ± Standard Deviation
Age (years)	28	44	29	27	36	33 ± 6
Body Mass Index (kg/m^2^)	20.2	26.4	23.5	23.8	20.9	23 ± 2
Height (cm)	165	165	170	160	168	166 ± 3
Weight (kg)	55	72	68	61	59	63 ± 6
Pelvic tilt from supine (°)						
Steep,	23,	19,	17,	24,	29,	22 ± 4,
Shallow	17	12	8	13	16	13 ± 3
Gel-pad Thickness (mm)						
Steep,	10.2,	N/A,	9.8,	9.7,	N/A,	9.8 ± 0.3
Shallow	9.8	N/A	9.8	9.3	10.0	
Membrane Bowing (mm)						
Steep,	10.4,	N/A,	8.6,	10.9,	N/A,	10.0 ± 1.3
Shallow	11.7	N/A	9.4	10.9	7.8	

#### 2.2.2. Patients

Five patient datasets were acquired after volunteer image acquisition began, as part of a recurrent gynaecological tumor clinical trial (NCT02714621, REC: 15/WM/0470) [3]. For treatment imaging datasets, patients were oriented into a clinically judged treatment position, with the tumor as close to the magnetic isocentre as possible. Because pretreatment diagnostic referral imaging was not available, the earliest (Day-7) follow-up supine images were used as ‘referral’ imaging datasets. These were chosen to minimize anatomical changes between the two imaging datasets. 15 mm-thick gel-pads were used for patients P2 to P5. For patient P1, a 40 mm-thick gel-pad was manually cut out to provide a degassed-water-filled recess, into which the patient was lowered. Patient details are recorded in Table 2. Weight data had been collected from patients as part of the trial data, but height data (and therefore BMI data) had not.

**Table 2:** Details of patients participating in this study.

Patient	P1	P2	P3	P4	P5	Mean ± Standard Deviation
Age (years)	64	53	72	74	59	64 ± 8
Weight (kg)	42	76	57	61	61	59 ± 11
Treatment Angle (°)	6	33	16	9	24	18 ± 10
Gel Pad Thickness (mm, mean ± SD)	5.3 ± 0.5	10.9 ± 0.6	8.6 ± 0.4	12.3 ± 0.4	8.0 ± 0.4	10 ± 2
(Nominal)	(40)	(15)	(15)	(15)	(15)	(15)
Membrane Bowing (mm, mean ± SD)	4.1 ± 0.2	10.0 ± 0.5	9.0 ± 0.5	5.0 ± 0.2	10.0 ± 0.1	7.6 ± 2.8

Patient referral and treatment imaging datasets were acquired after gadolinium contrast injection for improved contrast, and were acquired with a Field-of-View (FoV) of 288 × 288 × 133 voxels and voxel size 0.87 × 0.87 × 1.50 mm^3^. As part of a separate study, patient’s tumors were segmented from patient T2wLFOV datasets (TE = 90 ms, TR = 3620.4 ms, number of echoes = 16, flip angle = 90°, FoV 672 × 672 × 40 voxels, voxel size 0.45 × 0.45 × 4.5 mm^3^) obtained immediately pretreatment. These segments were used to define the target tumor volume for each patient.

### 2.3. Image registration

Registration of referral imaging datasets to treatment imaging datasets rotated the referral imaging dataset into the same treatment orientation as used in the treatment imaging dataset, which allowed the target coverage predicted from the registered-referral imaging dataset to be compared to that calculated from the treatment imaging dataset. Each subject’s referral imaging dataset was registered to their treatment imaging dataset(s) by aligning 10 or more manually placed bony landmark points, distributed throughout the pelvis, using Horos v2.4.0 (Horos Project) [7]. Registration was performed following the standard operating procedure described in the Appendix. The software calculated the required affine transformation and applied it to the referral imaging dataset [8] to generate the registered-referral imaging dataset.

To quantify the quality of this registration, the intra-observer (3 volunteer datasets) error and inter-observer (3 observers, 1 volunteer dataset) error associated with the referral-to-treatment registration was calculated. The errors were quantified as the mean Euclidean distance between corresponding points.

### 2.4. Image segmentation

The presence of acoustic obstructions and organs at risk in the beam path prevents safe sonication of the target, and hence they were segmented in order to identify acoustic access to the target. The tumor defined the target volume for patients, and hence was segmented. The body outline was segmented to assist with the other segmentation processes, and to assist in positioning the MRgHIFU system relative to the registered-referral imaging dataset. Organs at risk, bone (an acoustic obstruction) and the tumor were manually segmented from the MR datasets (as shown in Figure 2, Step 2). The body outline and extracorporeal air (an acoustic obstruction) were segmented automatically, as described below.

#### 2.4.1. Body outline

The body outline delineates the skin surface, and, particularly for treatment imaging datasets, needs to be separated from the gel-pad the subject lies on. An automatic process involving Otsu thresholding [9] was developed to separate the body from surrounding extracorporeal air and the gel-pad. Connected-components labeling [10] was used to collate segments of the body, and morphological operations [11] and flood-filling [12] were employed to link disparate segments and fill holes within segments.

#### 2.4.2. Acoustic obstructions

Internal acoustic obstructions, primarily bone, were segmented by manual contouring of axial slices using OsiriX Lite v10.0.4 [13] (Pixmeo, Geneva, Switzerland) and Horos. For volunteers, pelvic bones were manually segmented from referral imaging datasets. The registered-referral imaging dataset pelvic bone segments were applied to the corresponding treatment imaging dataset in order to reduce the burden of manual contouring. Femora were manually segmented separately from referral and treatment imaging datasets, because of the likelihood of different articulation between datasets (unlike the more rigid pelvis). For patients, the treatment region was considerably smaller and therefore pelvic bones as well as femora close to the target (tumor) could be manually segmented in a realistic time. However, contouring was restricted to ±10 axial slices from the edges of the tumor to reduce the time burden of manual segmentation. The pelvic bones at the greater sciatic notch were always segmented, because the notch defines the superior edge of the sciatic foramen through which the acoustic beam is expected to sonicate the tumor.

Air gaps between the patient and the gel-pad act as acoustic obstructions. Extracorporeal air in volunteer treatment imaging datasets was not segmented, because the trousers worn by volunteers during image acquisition prevented skin-to-gel-pad acoustic coupling. Instead, volunteer acoustic coupling limits in the left-right direction were manually identified, as shown in Figure 4. For volunteers, it was assumed that the intergluteal cleft would be filled with acoustic-coupling gel as part of clinical preparations, and hence, they were not treated as acoustic obstructions. Extracorporeal air in the patient treatment imaging datasets was segmented to define the limits of acoustic coupling, using an automatic segmentation algorithm inspired by Kullberg *et al.* [14]. In some cases, the intergluteal cleft was seen to contain air, and was therefore manually contoured and included as part of the extracorporeal air segment.

#### 2.4.3. Target volume

As part of a separate study, patient tumors had been contoured by an experienced radiographer (SG) using in-house software (Adept v0.2, The Institute of Cancer Research, UK) [3] on referral and treatment imaging T2wLFOV images, where the slice thickness was 10 times that of the in-plane voxel dimensions. Segmented tumors were registered to align with the Dixon imaging datasets using the same procedures described above in order to obtain tumor outlines in the Dixon images. Since healthy volunteers had no tumors, all soft tissue within the pelvic region was designated as the target.

#### 2.4.4. Organs at risk

Organs at risk, namely the uterus, rectum, bladder, and intestines were manually segmented for patients. Some patients had previously undergone pelvic exenteration surgery resulting in the removal of most pelvic organs.

#### 2.4.5. Evaluation of automated segmentation quality

Automatic segmentation quality for the body outline and for extracorporeal air was assessed by comparing randomly selected image slices with corresponding manually segmented slices (body: five slices per dataset, from three ‘steep’ treatment imaging datasets and two ‘steep’ registered-referral imaging datasets originating from three volunteers; air: five slices per dataset from three patient treatment datasets). In order to determine the ability of the segmentation to determine acoustic coupling between patient and transducer, only the extracorporeal air segments around the body/gel-pad interface were assessed.

The assumption that the manually-segmented pelvic bone in volunteer registered-referral datasets could be used to automatically segment the pelvic bones in the treatment imaging dataset was similarly tested against manual contouring performed on the treatment imaging dataset (five slices per treatment dataset, four treatment datasets originating from three volunteers). The segmentation quality of the volunteer bony pelvis and femora was taken to be indicative of the segmentation quality for all manually segmented tissues. Quality metrics were Dice Similarity Coefficient (DSC) and mean contour-to-contour distance [15,16].

### 2.5. Prediction of target volume coverage

#### 2.5.1. Overview

To calculate the target volume that can be covered, an MRgHIFU transducer was simulated. Positioning of the MRgHIFU transducer was known for the treatment imaging datasets, but had to be derived for the registered-referral imaging datasets. In the process of positioning the virtual transducer/referral imaging dataset, patient-induced compression of the gel-pad and bowing of the oil-bath membrane had to be taken into account. To reduce the computational time required, additional practical and clinically-relevant restrictions were placed on transducer translation, as described in greater detail below. The target volume covered by treatment cells was calculated for corresponding pairs of registered-referral and treatment datasets, and then, for each subject, the two volumes were compared. The details of these procedures are presented below.

#### 2.5.2. MRgHIFU system characteristics

**Figure 3.**
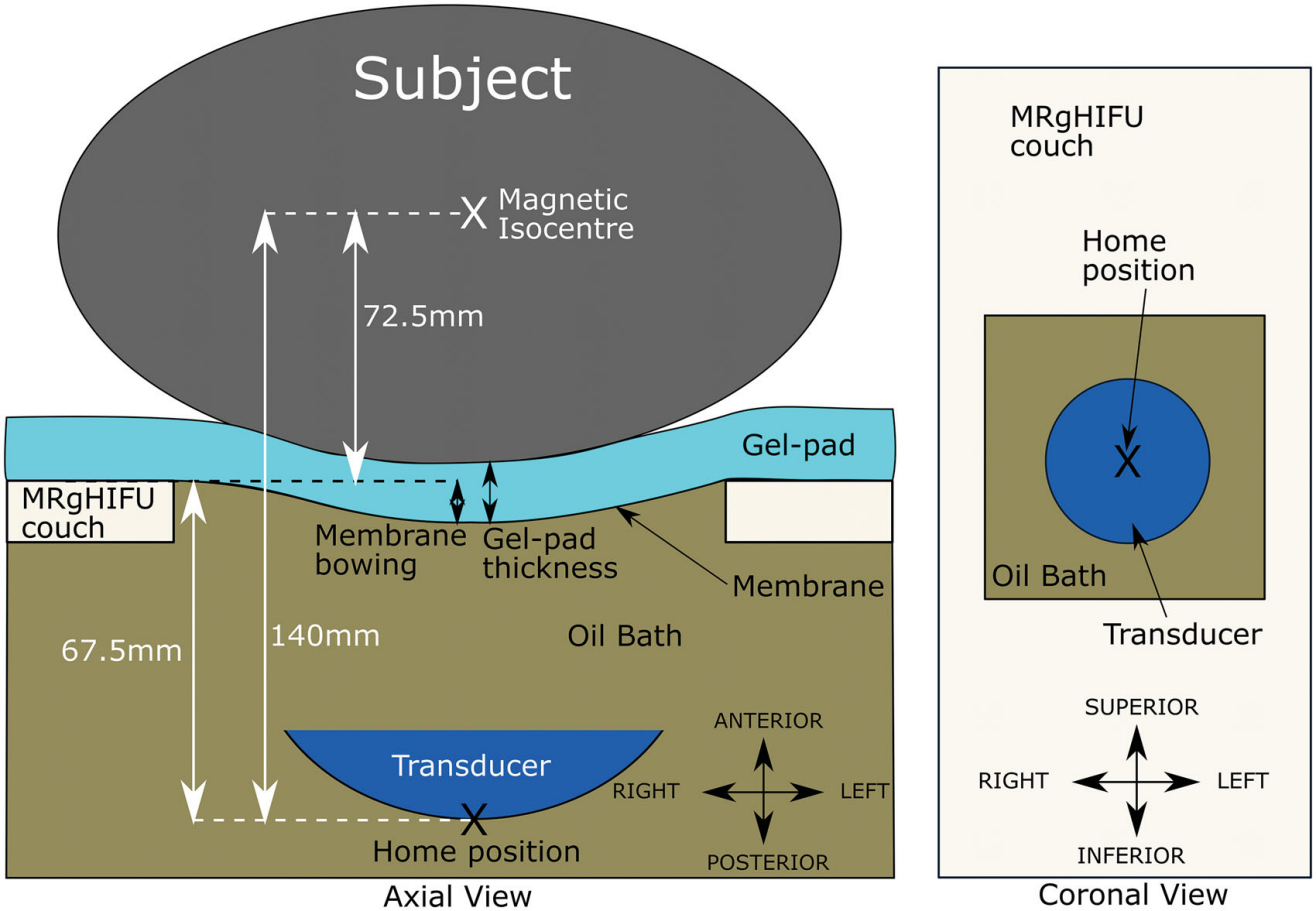
Schematic of the Sonalleve^®^ V2 MRgHIFU system: LEFT - a subject lying on the MR bed will compress the acoustic-coupling gel-pad and bow the acoustic membrane, which seals the oil bath. Ideally, target tissue would be centered directly above the transducer’s home position and the center of the membrane/gel pad and below the magnetic isocentre. RIGHT- a coronal view of the MRgHIFU couch showing the transducer’s home position below the center of the membrane.

The simulated transducer was modeled on The Royal Marsden Hospital’s MRgHIFU system, the Sonalleve^®^ V2. The system replaces the imaging couch in the bore of the MR scanner for treatment. The 256-element phased-array transducer (130 mm diameter, focal length 140 mm, source frequency 1.22 MHz) is mounted on a robotic positioner with 3 linear and 2 rotational motion capabilities in an oil bath, and faces the patient through a thin (50 µm thick) acoustically transparent membrane. The transducer’s home position (black cross in Figure 3) always lies 140 mm below the magnetic isocentre, and the undeformed membrane-to-isocentre distance is 72.5 mm. Acoustic coupling is achieved using a degassed-water wetted gel-pad (either 15 or 40 mm thick). When a subject is in place, the gel-pad is compressed and the acoustic membrane bowed under their weight. From its home position, the transducer can translate in 50 µm steps up to: 72.5 mm left or right and inferior or superior, and 34 mm toward the patient (anterior) and 33 mm away (posterior). The transducer can be angled up to 10° away from the perpendicular in the left-right and inferior-superior directions.

The transducer was simulated in MATLAB R2018b. It consisted of 256 points that represented the center of each transducer element. Ultrasound rays traced from each element on the transducer surface to the transducer focal point were used to represent the acoustic beam. The transducer was restricted to being able to tilt ±10° in 2.5° steps in the left-right direction only, in order to avoid incomplete registered-referral dataset image slices resulting from registration, but otherwise possessed the translational extents of the clinical device as described above. The transducer is assumed to produce a perfect 8 mm treatment cell, i.e. an 8 mm x 21.84 mm ellipsoid [17,18] centered at the focal point with its long-axis aligned to the beam axis.

#### 2.5.3. Practical and clinically-relevant restrictions on transducer translation

**Figure 4.**
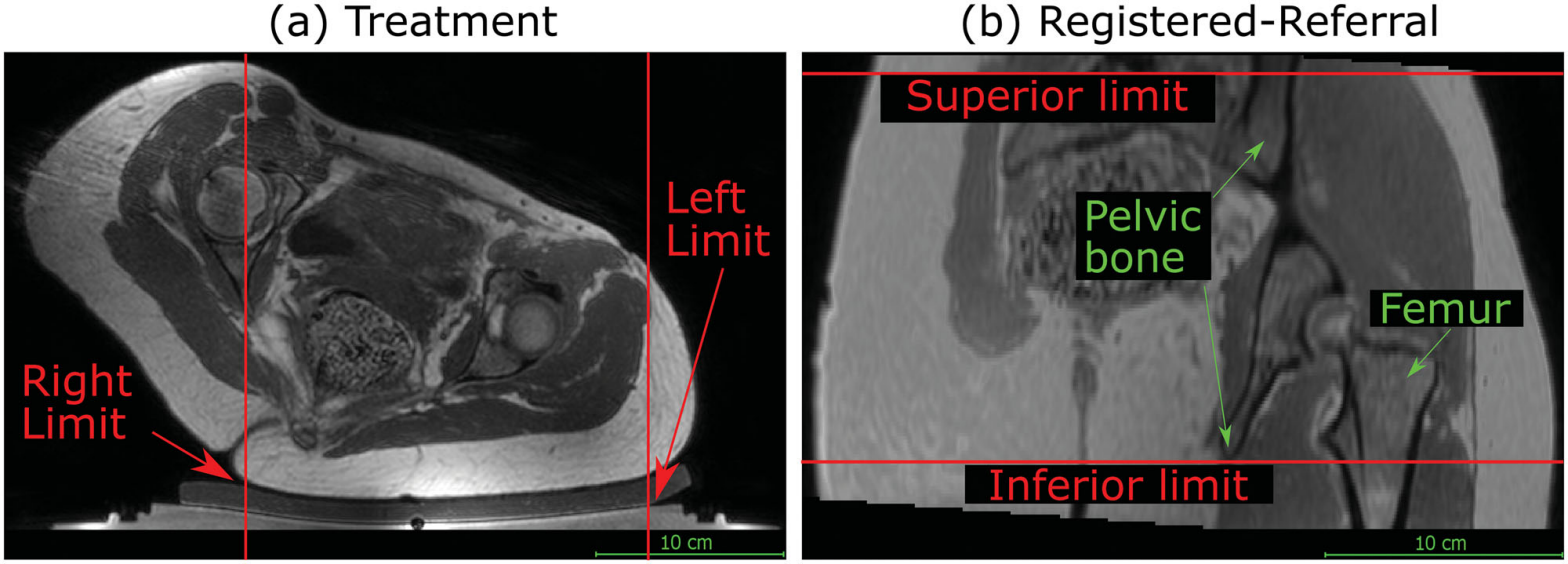
Transducer translation restrictions for volunteer data. Practical restrictions applied to the transducer’s translation capabilities (solid red lines) for volunteer datasets only. (a) For a treatment imaging dataset, the left-right translation was limited by the extent of acoustic coupling between the volunteer’s skin and the gel pad. The corresponding registered-referral imaging dataset shared these left-right restrictions. (b) For a registered-referral imaging dataset, the transducer’s inferiorsuperior translation was restricted by the extent of pelvic bone and the requirement for a full body outline within the image. The corresponding treatment imaging dataset shared these inferior-superior restrictions.

In order to improve computational efficiency of target coverage prediction, transducer translation in the left-right and inferior-superior axes was restricted to the left-right and inferior-superior extents of the targets. For patients, practical restrictions on left-right and inferior-superior translation were calculated from the left-right and inferior-superior extents of the tumor. For volunteers, the target is all soft tissue within the pelvic region. Hence, practical and clinically-relevant limits were manually identified (see Figure 4) and implemented. The left-right limits represent the extents of acoustic coupling. The inferior-superior limits represent the inferior-superior extents of the registered-referral imaging dataset containing complete body outlines and pelvic bone.

#### 2.5.4. Estimated patient deformation resulting from reorientation into the treatment position

**Figure 5.**
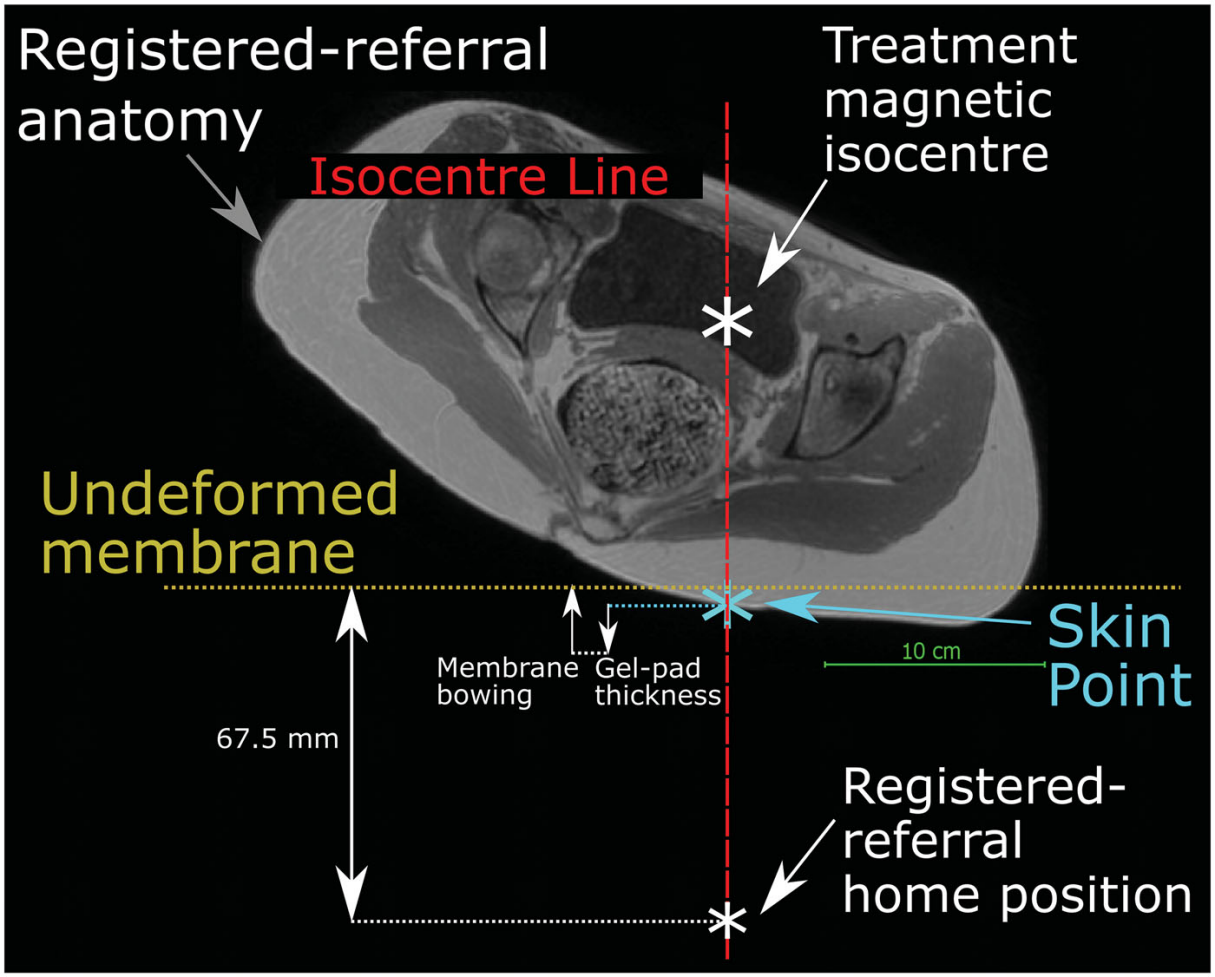
Method used to predict the transducer’s anterior-posterior home position in a registered-referral imaging dataset. The treatment dataset magnetic isocentre is known because the registered-referral imaging dataset had been registered to the treatment imaging dataset. A line was drawn downwards from the treatment dataset isocentre and intersected the skin at the skin point. From this skin point, the home position was calculated using the average compressed gel-pad thickness, the average membrane bowing distance, and the calibrated distance between undeformed membrane and home position of 67.5 mm (see Figure 3).

In this study, the treatment position was known from the treatment imaging dataset. In treatment imaging datasets, the isocentre, and hence the transducer’s home position (Section 2.5.2), was known. In the registered-referral imaging dataset, because the treatment position is the same, the transducer’s home position left-right and inferior-superior coordinates were taken from the treatment imaging dataset. However, to mimic the prospective workflow, the anterior-posterior coordinate had to be estimated from data within the registered-referral imaging dataset. The method of doing so is shown in Figure 5. Briefly, it was assumed that: i) the gel-pad would be most compressed and the membrane most bowed at the isocentre line, and ii) after soft tissue deformation resulting from the reorientation into the treatment position, the isocentre-to-skin point distance would remain the same. The membrane bowing distance and gel-pad thickness for patients was assumed to be that calculated for volunteers. These quantities were obtained by determining the average gel-pad thickness and membrane bowing distance close to the isocentre line, using ITK-Snap, in the 7/10 volunteer treatment imaging datasets in which measurement was possible. From this, the position of the undeformed membrane, and hence the transducer anterior-posterior home position, was estimated (see Figure 3). Patient P1 had been treated on a customized gel-pad, the thickness of which was independently measured and used for positioning. For comparison, the actual patient gel-pad thicknesses and membrane bowing distances were measured and compared to the volunteer-derived averages.

#### 2.5.5. Calculation of target coverage

**Figure 6.**
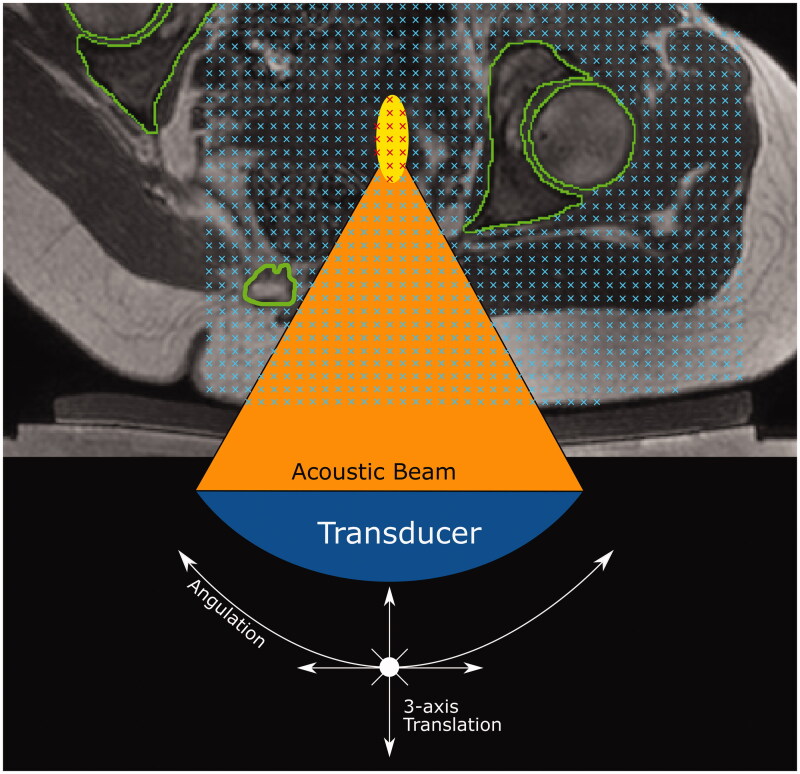
Method for quantifying target volume covered within a dataset (volunteer treatment imaging dataset in this example). A regular 3D grid of potentially accessible points was created (blue crosses) within the target: soft tissue (volunteers) or tumor (patients). For each transducer position and tilt identified in Section 2.5.3, the acoustic beam was checked for intersection with any acoustic obstructions (green contours) or organs at risk. If no obstruction exists, an 8 mm treatment cell was created around the focus (yellow ellipse). Grid points within a treatment cell were marked as ‘covered' (red crosses).

For volunteers, a regular grid of target points, one per image voxel, was created in the soft tissue (see Figure 6); for patients, this grid was created solely within the tumor [19]. The transducer acoustic beam had been discretized into 256 rays, linking the center of a transducer element to the focus. Each ray was discretized into regularly spaced (0.2 mm) points along its length, and each was tested for intersection with acoustic obstructions or organs at risk. If no point intersected these, an 8-mm treatment cell was drawn around the focal point, and all grid points within this were marked as covered (Figure 6). This was repeated as the transducer was exhaustively translated and tilted. The number of grid points covered, multiplied by the image voxel volume, was used to quantify the target volume covered. For volunteers, the transducer was translated in 4 mm steps, whereas for patients, 2 mm steps were used in order to ensure coverage of the smaller tumor volume.

For volunteers, the accuracy of the methodology was quantified by calculating how much of the soft tissue volume coverage calculated from the treatment imaging dataset was predicted to be covered from the registered-referral imaging dataset, as described in Equation (1). In effect, the treatment imaging dataset covered soft tissue volume becomes the target volume for the registered-referral imaging dataset, allowing calculation of the percentage target volume covered (TVC_vol_).

(1) $$TVC_{\mathrm{vol}}=100\%\times \frac{CV_{\mathrm{RegisteredReferral}}\cap CV_{\mathrm{Treatment}}}{CV_{\mathrm{Treatment}}}$$

where CV is the covered target volume.

For patients, the accuracy of the methodology was quantified using the difference between the percentage tumor volumes covered (TVC_pat_), calculated from treatment imaging dataset and that calculated from registered-referral imaging dataset. TVC_pat_ is given by:

(2) $$TVC_{\mathrm{pat}}=100\%\times \frac{CV}{TV}$$

where CV is the covered tumor volume and TV is the total tumor volume.

## 3. Results

### 3.1. Subjects

Details for the volunteers involved in the study are recorded in Table 1, and those for patients in Table 2, as are the (pseudo-)treatment angle(s), compressed gel-pad thickness and membrane bowing distance for each subject. For volunteers, 15 mm gel-pads were compressed to an average of 9.8 ± 0.3 (mean ± standard deviation, with range: 9.3 to 10.2) mm, and the average membrane bowing distance close to the isocentre line was 10.0 ± 1.3 (range: 7.8 to 11.7) mm. The weight ranges of volunteers and patients (patients: 59 ± 11 kg vs volunteers: 63 ± 6 kg) were similar. The range of patient treatment angles (6-33°) slightly exceeded the range of volunteer angles (8-29°).

### 3.2. Image registration quality

Between three observers, the mean distance between corresponding points for the referral imaging dataset for one volunteer, registered to one of their treatment imaging datasets, was on average 1.2 ± 0.2 mm. For one observer, the mean distance between corresponding points for the referral imaging datasets for three volunteers, each registered to one of their corresponding treatment imaging datasets, was on average 1.3 ± 0.2 mm. These distances are less than the axial slice thickness of the Dixon image datasets and less than double the in-plane image resolution.

### 3.3. Segmentation quality

#### 3.3.1. Automatic segmentation quality

Automatically segmented body outlines agreed with validation slices with a mean DSC of 0.991 ± 0.003 and an average mean contour-to-contour distance of 0.9 ± 0.4 mm. Automatic extracorporeal air segmentation of patient data agreed with validation slices with a mean DSC of 0.89 ± 0.06 and an average mean contour-to-contour distance of 0.25 ± 0.16 mm.

#### 3.3.2. Manual segmentation quality

Volunteer treatment image pelvic bone segmentation agreed with the validation slices, with mean DSC of 0.93 ± 0.01 and an average mean contour-to-contour distance of 0.76 ± 0.10 mm. Volunteer femur segmentation agreed with the validation slices with mean DSC of 0.96 ± 0.01 and an average mean contour-to-contour distance of 0.53 ± 0.11 mm.

### 3.4. Prediction of target volume coverage

**Figure 7.**
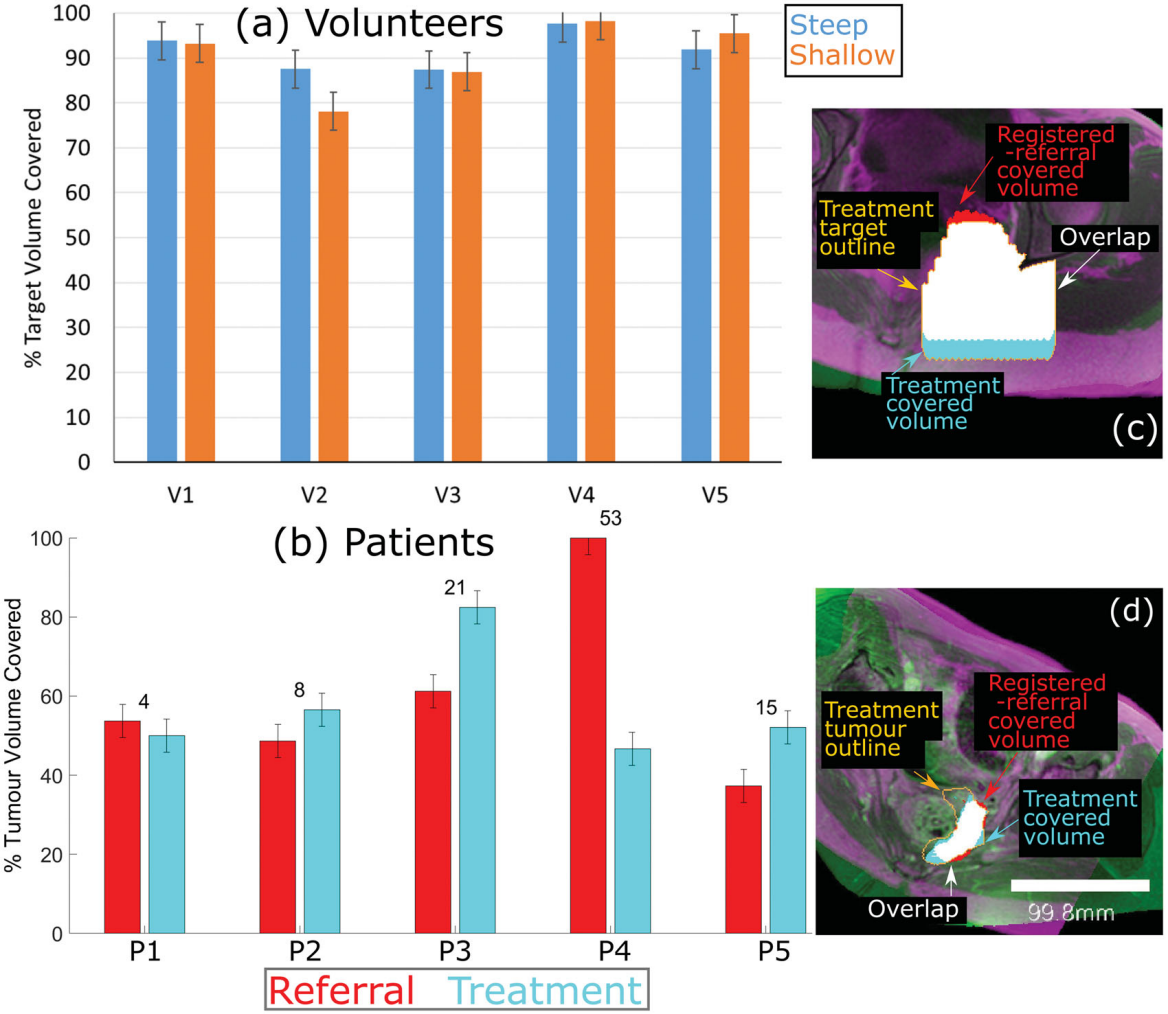
Percentage of target volume covered. (a) For volunteers, the agreement between the referral and treatment covered volumes is shown, where the treatment covered volume is the ground-truth. (b) For patients, the percentage of the registeredreferral tumor (red) and the treatment tumor (blue) that was covered is shown. The numbers on top of each set of bars represent the difference in % Tumor Volume Covered predicted from the registered-referral dataset, and that calculated from treatment dataset. Representative examples of target coverage for volunteers (c) and tumor coverage for patients (d) are shown, with a scale bar in (d). The anatomy is shaded purple in the registered-referral dataset, and green in the treatment dataset.

The TVC_vol_ for each volunteer in each of their two treatment positions is shown in Figure 7(a). For volunteers, the registered-referral imaging dataset predicted target volume coverage of 91 ± 6% (range: 78 to 98%) of that calculated from the corresponding treatment imaging dataset. The TVC_pat_ for each patient’s treatment imaging and referral imaging are shown in Figure 7(b). Patient P4 appears to be an outlier. Excluding their data, for patients, registered-referral TVC_pat_ predicted the treatment TVC_pat_ to within an average of 12 ± 7% (range: 4 to 21%). Representative images of the target (tumor) volumes covered for volunteers and patients are shown in Figure 7(c) and (d), respectively.

## 4. Discussion

The aim of this study was to develop a novel method to calculate tumor coverage and assess the feasibility of predicting tumor coverage from (supine) referral imaging, as part of a wider study into automating the evaluation of patient suitability for MRgHIFU therapy.

### 4.1. Subjects

Although patient mean age was nearly double that of the volunteers, their weights were similar. Compressed gel-pad thickness and membrane bowing for volunteers varied minimally (mean ± standard deviation being 9.8 ± 0.3 mm and 10.0 ± 1.3 mm respectively), suggesting that use of mean values for the prediction of patient tumor coverage should be acceptable. Minimum and maximum patient tilt angles exceeded those of volunteers by at most 4° despite acquiring the volunteer imaging before the patient data was available.

### 4.2. Image registration

Mean post-registration misalignment between referral and treatment images was found to be less than the axial slice thickness of the Dixon MR imaging, in line with results from literature [20].

### 4.3. Image segmentation

Automatic and manual segmentation of acoustic obstructions, organs at risk and the body outline resulted in mean DSCs ≥ 0.89 and mean contour-to-contour distances that were less than the axial slice thickness (1.5 mm). A mean contour-to-contour distance of 2.81 mm has been deemed acceptable for breast-air boundary segmentation from MR imaging (voxel size: isotropic 2.5 mm) [21]. The DSC for extracorporeal air segmentation (patient treatment imaging datasets only) was less than that for body outline segmentation (volunteers and patients, treatment and registered-referral datasets) while the mean contour-to-contour distance was better than that for body outline segmentation. This was probably due to the smaller size of the air segments around the patient/gel-pad interface, causing a misidentified voxel to have a greater effect than for the larger body outline. From the DSC (0.96) and mean contour-to-contour distance (0.53 mm) values, the assumption that pelvic bone segments identified on treatment images were identical to post-registration, manually outlined referral image segments appears to be valid (Section 3.3.2).

Since tumors were manually segmented by an expert, any segmentation imprecision or inaccuracy was ignored. Tumors were segmented on datasets with slice thickness (4.5 mm) 10 times the in-plane resolution (0.45 mm), and thus rotation during registration could introduce relatively large discrepancies between the interpolated and actual tumor outlines, thus increasing uncertainty in the TVC_pat_ predicted from referral imaging datasets.

### 4.4. Target coverage

#### 4.4.1. Volunteer study

The volunteer’s results show an average target coverage agreement between treatment and registered-referral imaging datasets of 91% (range: 78–98%), corresponding to a mean difference of 9%. This suggests that the techniques used for positioning the transducer in the registered-referral imaging datasets were sufficient to proceed to testing with patient data. The worst agreement (78%, for Volunteer 2 tilted at a 12° treatment angle) was attributed to inaccurate placement of the transducer’s home position, caused by the skin point directly below the isocentre (see Figure 5) not remaining at constant position between the registered-referral and treatment datasets, as had been assumed. Consequently, the HIFU focus was predicted to reach 12 mm deeper into the volunteer than it could. The next worst agreements, (88% for Volunteer 2 tilted at 19° and Volunteer 3 tilted at 17° and 8°) were due to the same cause, resulting in overestimation of the focal depth by 6 mm.

For a single volunteer, the difference between target coverage predicted from registered-referral datasets and that from treatment datasets results from differing femur segments and differing transducer home positions. Since angulation was restricted to tilting left-right only, and the transducer was restricted to prevent translation beyond the inferior-superior extents of the pelvis, differences in femur segmentations were judged to have only a small effect. Refinement of the transducer positioning technique, by sampling within a 15 × 15 mm region around the isocentre line (see Figure 5) instead of using a single skin position to predict the anterior-posterior position, provided no statistically significant improvement (data not presented).

#### 4.4.2. Patient study

The goal of this study was to develop and test a method for quantitatively assessing tumor coverage from referral imaging, as opposed to the current clinical practice of qualitative assessment, and to assess the feasibility of the new methodology. From the results, quantitative prediction of tumor coverage from referral imaging appears feasible. Despite the simplicity of the technique used to account for the expected body deformation resulting from reorientation from supine into a treatment position, the TVC_pat_ predicted from the registered referral and the treatment imaging datasets had a mean difference of 12% (range: 4–21%), excluding an outlier for whom the difference was 53% (see below). In the literature, a median difference of 21% in automatic segmentation had been judged as acceptable [22]. In the context of the current clinical practice, where ≥40% of referred patients fail screening, these results are encouraging [3,4]. The small cohort involved in this study (5 volunteers, 5 patients) represents lower than expected patient recruitment for the clinical trial. However, other published studies have also involved small patient cohorts, e.g. a transcranial simulation study involved 5 patients [23], a simulation study for kidney ablation examined 4 patients [24], and in various therapeutic feasibility studies, between 10 and 13 patients were considered [25–27]. In addition, an automatic geometric optimization technique for the packing of HIFU treatment cells demonstrated its capabilities using test objects and the publicly available dataset of a single volunteer [28]. Results from these small-cohort feasibility studies also demonstrate high variance in results. For example, in the transcranial simulation study, simulation results differed from measured data by up to 40 ± 13% [23]. The results here indicate a step toward the long-term objective of widespread quantitative analysis of patient suitability for MRgHIFU therapy, with the aim of improving clinical decision-making and minimizing the impact on patient and hospital time and resources.

The outlier referred to above was patient P4, whose poor results were due to the assumption of perfect acoustic coupling between patient and gel-pad when calculating TVC_pat_ for the registered-referral imaging dataset. In practice, treatment imaging showed that the tumor periphery was obstructed by air between the patient and gel-pad. This highlights a possible advantage of the proposed workflow. Having established that a greater tumor coverage could have been achieved at the referral stage, clinicians may have been able to improve the clinical preparations, and increase tumor coverage.

In general, the marginally poorer results for patients compared to volunteers (excluding the outlier patient) may be partially due to volunteer target volumes being over 10 times larger (∼300,000 ± 100,000 mm^3^) than patient targets (∼20,000 ± 10,000 mm^3^). A missed voxel has a larger proportional effect for smaller target volumes.

A source of error for the patient cohort may arise from the differences in the actual gel-pad thickness and membrane bowing (Table 2) compared to the mean values determined from the volunteer cohort which were used in the predictive calculations. Membrane bowing differences from the average of 10.0 mm ranged from 0.9 mm to 4.7 mm for patients, and from 0.4 mm to 2.2 mm for volunteers. Gel-pad thickness differences from the average of 9.8 mm ranged from 1.1 to 2.5 mm for patients who were treated on 15 mm gel-pads, and from 0.0 to 0.7 mm for volunteers. To evaluate the effect of this, the TVC was recalculated with the actual gel-pad thickness and membrane bowing distance for all patients. The maximum difference in TVC_pat Registered-Referral_ that resulted from using the average membrane bowing and gel-pad thickness, rather than the actual measured values, was 0.3% (patient P1). As more data from clinical studies becomes available, modeling the relationship between membrane bowing distance, or compressed gel-pad thickness, and patient weight and orientation may generate more accurate predictions of the transducer home position from referral imaging.

Deformation and translation of organs at risk, due to reorientation from referral to treatment position, clinical preparation such as pretreatment dieting and bowel-preparation, and the time between referral and treatment (1 week), may explain why the patient results show worse agreement overall than the volunteer results. In clinical experience, organs at risk such as the rectum are known to vary substantially and unpredictably in shape, position and volume [29,30]. The overall accuracy of the proposed patient workflow is expected to be limited by the patient-specific soft tissue deformation and coupling to the gel-pad. At the very least, the methodology presented here allows quantitative assessment of tumor coverage prior to the screening stage, reducing the need for clinical experience, and the influence of subjective opinion, on the assessment of patient suitability for progression through the treatment pathway.

### 4.5. Limitations of the study

One of the major limitations is the small volunteer and patient cohort, which restricts the statistical certainty of the results. This study is also limited to predicting pelvic tumor coverage. However, the proposed patient workflow may be adaptable for other tumor sites. Assessment of the tumor volume that can be successfully ablated will require acoustic propagation and thermal bioeffects modeling. This is the subject of extensive ongoing work. Patient deformation resulting from orientation into the treatment position was only accounted for using the simple assumption that the isocentre-to-skin point distance would remain constant. This produced acceptable results for tumor coverage. However, accurate acousto-thermal modeling requires an accurate description of the medium of propagation, which may require simulation of soft tissues deformation between the gel-pad and the target.

Only reorientations from supine to oblique supine decubitus positions were tested in this study. While the results of this study are only applicable to the specific diagnostic MR bed and MRgHIFU couch used, the core principles are expected to be applicable to other HIFU devices, and referral datasets obtained from X-ray tomographic imaging. Furthermore, since the patient mean age was almost twice that of the volunteers, patient soft tissue could have different elastic properties than that of volunteers and therefore exhibit different deformation behavior. This could have affected the developed methodology.

## 5. Conclusion

Novel methodology for predicting the MRgHIFU target coverage from supine (MR) referral imaging was developed using 10 volunteer datasets and was retrospectively applied to 5 patient datasets. The difference between the target coverage computed using referral and treatment image datasets was within 12% on average (range: 4–21%), after one patient, with inadequate acoustic coupling during treatment, was excluded from analysis. Despite the relatively small cohort size, the focus on pelvic tumors, and the limited range of patient positions and MRgHIFU equipment on which the methodology was devised and tested, these results suggest quantitative, automated screening and treatment planning should be feasible, eventually obviating the need for patient suitability to be assessed using qualitative clinical judgment based on operator experience.

## Geolocation information

The study was conducted at the Institute of Cancer Research and the Royal Marsden Hospital, Sutton, Surrey, United Kingdom.

## Data Availability

The data that support the findings of this study are not available due to limitations in the ethical review.

## Appendix 1 Appendix: *Registration standard operating procedure*

1. Open Horos™ on Mac OS X. Make sure the pyOsiriX plugin is installed.
2. Import the in-phase MRI datasets that are to be registered. Double click them to bring them up together.
3. Select a dataset. Then, at the top menu 2D Viewer → Sort By…→ Slice Location Ascending.
4. Below the menu bar, in a section titled ‘Mouse button function’, select the point function. Use the point function to mark an anatomical feature on one dataset and the same anatomical feature on the other. The same point names, e.g. ‘Point 1’, must correspond to the same anatomical features in both datasets. Repeat this for the list of anatomical features mentioned below. If the same anatomical feature cannot be found in one or both of the datasets, ignore that anatomical feature and continue down the list. At least 10 features should be marked by the end.
  Anatomical Features:
  - Femur/pelvis landmark marks where the two bones meet in the head-most direction (Right and Left)
  - ischial spine (Right and Left)
  - Superior-most or inferior-most of ischial tuberosity (Right and Left)
  - Pubic arch/top of pubic arch connection
  - Anterior-facing spur in axial plane where pelvis first encloses femur head (Right and Left)
  - Sacral nerve bundle (S1 and S2) when just-enclosed by bone (Right and Left)
  - Spinal nerves splitting from spinal cord (Right and Left)
  - Sacrum/L5 disk border
  - Coccyx
5. Open the pyOsiriX console within Horos. A Python script can be used to extract point data from a dataset in Horos and save it in a format that can be processed in an external Python environment. Do this for both datasets.
